# The associations of hepatic steatosis and fibrosis using fatty liver index and BARD score with cardiovascular outcomes and mortality in patients with new-onset type 2 diabetes: a nationwide cohort study

**DOI:** 10.1186/s12933-022-01483-y

**Published:** 2022-04-16

**Authors:** Jiyun Park, Gyuri Kim, Bong-Sung Kim, Kyung-Do Han, So Yoon Kwon, So Hee Park, You-Bin Lee, Sang-Man Jin, Jae Hyeon Kim

**Affiliations:** 1 Division of Endocrinology and Metabolism, Department of Medicine, Samsung Medical Center, Sungkyunkwan University School of Medicine, 81, Irwon-ro, Gangnam-gu, Seoul, 06351 Republic of Korea; 2 Department of Statistics and Actuarial Science, Soongsil University, 369 Sangdo-ro, Dongjak-gu, Seoul, 06978 Republic of Korea; 3 Department of Clinical Research Design and Evaluation, Samsung Advanced Institute for Health Sciences and Technology, Seoul, Republic of Korea

**Keywords:** BARD score, Cardiovascular disease, Fatty liver index, Mortality, New-onset type 2 diabetes

## Abstract

**Background:**

Although both type 2 diabetes mellitus (T2DM) and nonalcoholic fatty liver disease (NAFLD) are associated with increased risk of cardiovascular disease (CVD), evidence is lacking as to whether the presence of NAFLD confers an additional risk of CVD in patients with T2DM. We investigated the associations between hepatic steatosis and/or fibrosis and risk of myocardial infarction (MI), stroke, heart failure (HF), and mortality in patients with new-onset T2DM.

**Methods:**

Using the Korean National Health Insurance dataset, we included 139,633 patients diagnosed with new-onset T2DM who underwent a national health screening from January 2009 to December 2012. Hepatic steatosis and advanced hepatic fibrosis were determined using cutoff values for fatty liver index (FLI) and BARD score. Hazard ratios (HRs) and 95% confidence intervals (CIs) were estimated using multivariable Cox proportional hazards regression models.

**Results:**

During the median follow-up of 7.7 years, there were 3,079 (2.2%) cases of MI, 4,238 (3.0%) cases of ischemic stroke, 4,303 (3.1%) cases of HF, and 8,465 (6.1%) all-cause deaths. Hepatic steatosis defined as FLI ≥ 60 was associated with increased risk for MI (HR [95% CI], 1.28 [1.14–1.44]), stroke (1.41 [1.25–1.56]), HF (1.17 [1.07–1.26]), and mortality (1.41 [1.32–1.51]) after adjusting for well-known risk factors. Compared to the group without steatosis, the group with steatosis and without fibrosis (BARD < 2) and the group with both steatosis and fibrosis (BARD ≥ 2) showed gradual increased risk for MI, stroke, HF, and mortality (all *p* for trends < 0.001).

**Conclusion:**

Hepatic steatosis and/or advanced fibrosis as assessed by FLI or BARD score were significantly associated with risk of CVD and mortality in new-onset T2DM.

**Supplementary Information:**

The online version contains supplementary material available at 10.1186/s12933-022-01483-y.

## Background

Nonalcoholic fatty liver disease (NAFLD) is a metabolic liver disease that is strongly associated with insulin resistance and obesity. NAFLD is one of the important risk factors for type 2 diabetes mellitus (T2DM) and is also a consequence of T2DM; they commonly co-exist, and their relationship is considered bidirectional [1]. NAFLD is more prevalent in patients with T2DM (40–70%) compared to 10–30% in the general population [2, 3]. Although the mechanism remains unknown, patients with T2DM are more susceptible to adverse hepatic outcomes including fibrosis, cirrhosis, and hepatocellular carcinoma [4]. Therefore, the presence of NAFLD should be considered in patients with T2DM since they are at high risk for disease progression [5]. Because both NAFLD and T2DM contribute to cardiovascular disease (CVD) and mortality [6–8], it is possible that co-existent NAFLD and T2DM is associated with a higher CVD risk. However, few studies have evaluated the effect of NAFLD on CVD in T2DM. Therefore, the association between NAFLD and CVD independent of diabetes has not been determined. Furthermore, whether the population with hepatic steatosis and/or fibrosis at the time of diagnosis of T2DM has higher rates of CVD or mortality has not been evaluated.

Liver biopsy and imaging studies are the gold standard diagnostic tools for hepatic steatosis and fibrosis. However, because they are not available or feasible for use in large epidemiological studies, non-invasive assessments are preferred in these settings to identify hepatic steatosis and fibrosis [5]. Fatty liver index (FLI) and BARD score are biomarkers for predicting hepatic steatosis and fibrosis, respectively, and their associations with CVD have been validated in previous studies including the general population [2, 9, 10]. Several studies evaluating the association between FLI and CVD included patients with and without diabetes. Despite subgroup analysis stratified by diabetes, it is unknown whether the association between FLI and CVD for those patients is independent of diabetes because duration and therapy for diabetes were not considered. In addition, no longitudinal study has investigated the association between hepatic fibrosis using BARD score and CVD or mortality in newly diagnosed T2DM as a large-scale nationwide study.

In this study, we evaluated the associations of hepatic steatosis assessed by FLI and/or fibrosis assessed by BARD score with the risk of incident myocardial infarction (MI), stroke, heart failure (HF), and mortality in new-onset T2DM patients.

## Methods

### Data source and study population

**Fig. 1:**
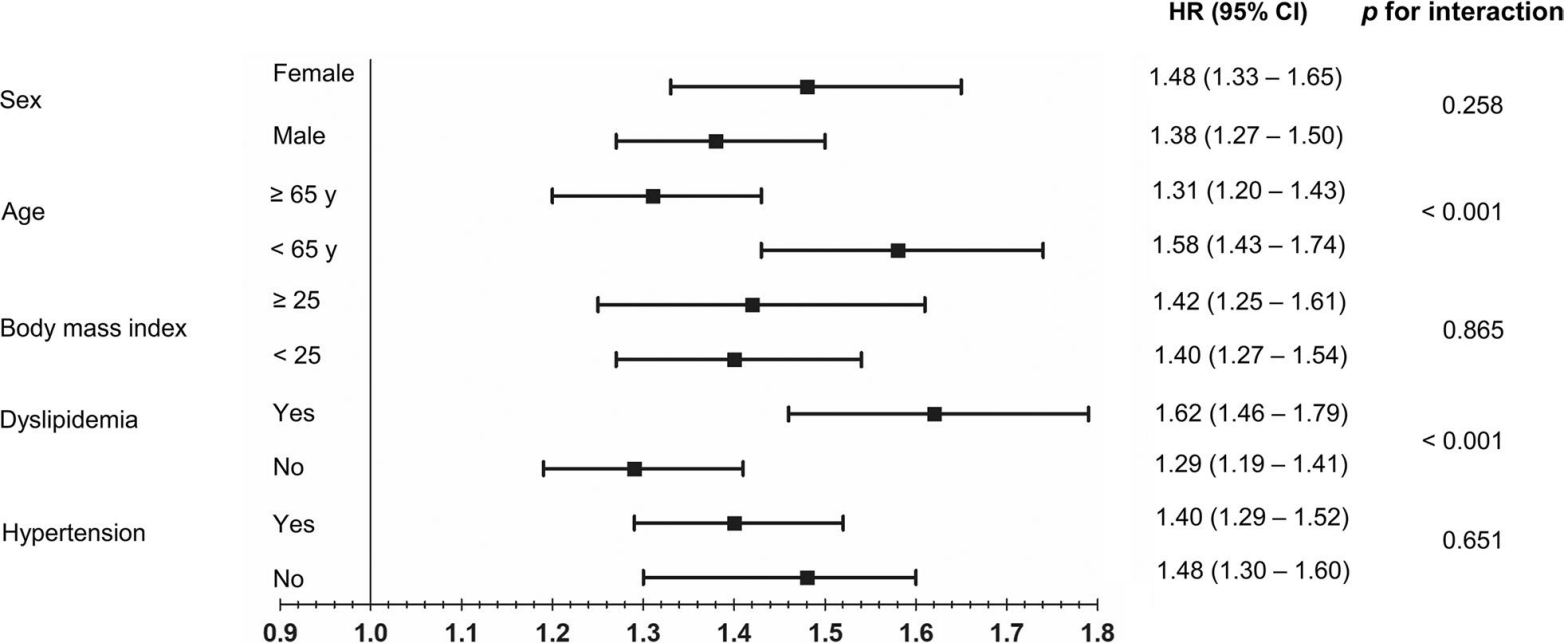
Subgroup analysis for mortality in patients with NAFLD (fatty liver index ≥ 60) compared to those without NAFLD (fatty liver index < 30). CI, confidence interval; HR, hazard ratio; NAFLD, non-alcoholic fatty liver disease

In this study, we used Korean National Health Insurance (NHIS) datasets of claims and preventive health check-ups in Korea from January 2009 to December 2012. The claims dataset offers information about diagnosis statements based on International Classification of Disease, 10th revision (ICD-10) codes and prescriptions. The South Korean government manages a public medical insurance system and provides a health check-up every two years, along with questionnaires on lifestyle and behaviors including alcohol consumption. Anthropometric and laboratory measurements are incorporated into the health check-up dataset. Detailed methods regarding these measurements were described in previous research [11, 12]. Among the individuals who underwent a health examination between 2009 and 2012, we included only patients with new-onset T2DM who started taking diabetic medication within one year of their health checkup [13]. T2DM was defined as at least one claim per year using ICD-10 codes E11 to E14 and at least one claim per year for prescription of antidiabetic medication or by a fasting glucose level of at least 126 mg/dL. Among 2,746,079 patients with T2DM between 2009 and 2012, individuals younger than 20 years and those who had a prescription record for insulin and/or at least one oral hypoglycemic agent prior to the health examination were excluded. Only the patients who initiated diabetic medication within one year of their health examination under the ICD-10 codes E11-14 were included. Among the 251,359 newly diagnosed T2DM patients, 111,726 with the following criteria were excluded: hepatitis or liver disease other than NAFLD; consumed alcohol ≥ 30 g/day; MI, stroke, HF, any cancer, rheumatic mitral valve disease, or cardiac/vascular implants or grafts; or missing data. Finally, 139,633 patients were included in our study (electronic supplementary material [ESM] Fig. 1). This study was approved by the Institutional Review Board of Samsung Medical Center (Approval No. SMC 2021-09-102), Seoul, Republic of Korea, who granted an exemption to the need for informed consent because all data provided by the NHIS to researchers were de-identified.

### Measurements and definitions of variables

Information on current smoking, alcohol consumption, and regular exercise was collected from self-reported questionnaires. Smoking was defined as never-smoker, former-smoker, or current-smoker. Alcohol history was classified as none, mild drinker < 30 g of alcohol/day, or heavy drinker ≥ 30 g of alcohol/day [14, 15]. Regular exercise was defined as high intensity of activity performed for at least 20 min at least three times per week or moderate intensity of activity performed for at least 30 min at least five times per week [16]. Low income level was regarded as the lowest 20% of the total population based on monthly income [17]. Body mass index (BMI) was defined as weight in kilograms divided by the square of height in meters (kg/m^2^). Oral hypoglycemic agents included metformin, sulfonylurea, meglitinides, thiazolidinedione, dipeptidyl peptidase-4 inhibitor, and α-glucosidase inhibitor. Hypertension was defined as at least one claim per year using ICD-10 codes I10 or I11 and at least one claim per year for prescription of antihypertensive agents or by a systolic/diastolic blood pressure of at least 140/90 mmHg. Dyslipidemia was defined as at least one claim per year using ICD-10 code E78 and at least one claim per year for prescription of a lipid-lowering agent or by a total cholesterol level of at least 240 mg/dL [11].

### Definitions of hepatic steatosis and advanced hepatic fibrosis

FLI, used to define hepatic steatosis, was calculated according to the following equation: (e^0.95 × log^_e_^(triglyceride) + 0.139 × BMI + 0.718 × log^_e_^(gamma−glutamyl transferase) + 0.053 × waist circumference − 15.745^)/(1 + e^0.95 × log^_e_^(triglyceride) + 0.139 × BMI + 0.718 × log^_e_^(gamma−glutamyl transferase) + 0.053 × waist circumference − 15.745^) × 100. The subjects were classified into three groups: FLI ≥ 60, NAFLD; 30–59, intermediate group; and FLI < 30, no NAFLD [18]. Among patients with NAFLD (FLI ≥ 60 points), the presence of advanced liver fibrosis was defined based on the BARD score, which was calculated by assigning points for the presence of an AST/ALT ratio ≥ 0.8 (two points), BMI ≥ 28 kg/m^2^ (one point), and DM (one point), where a total score of two to four points indicates advanced hepatic fibrosis [19].

### Definitions of CV events

The study endpoint was development of MI, ischemic stroke, HF, or mortality. MI was defined as hospitalization with an ICD-10 code I21 or I22, and ischemic stroke was defined as ICD-10 code I63 or I64 during hospitalization with claims for brain imaging such as magnetic resonance imaging or computed tomography [9, 20]. HF was defined as hospitalization with ICD-10 code I50. The study population was followed from baseline to date of death or cardiovascular event or until December 31, 2018, whichever came first.

### Statistical analysis

Clinical and demographic characteristics are presented as numbers and percentages for categorical variables and means ± standard deviations or medians (interquartile ranges) for continuous variables. The event rate of the primary outcome is presented per 1000 person-years, determined by dividing the number of events by the total person-year period. Hazard ratios (HRs) and 95% confidence intervals (CIs) for the association between FLI or BARD score and CV outcome or mortality were analyzed using Cox proportional hazards analysis. Model 1 was adjusted for age and sex, and model 2 was additionally adjusted for smoking status, alcohol consumption, physical activity, income, body weight, hypertension, dyslipidemia, fasting glucose, and number of oral hypoglycemic agents used. Stratified analyses were performed according to age (< 65 years vs. ≥ 65 years), sex, BMI (< 25 kg/m^2^ vs. ≥ 25 kg/m^2^), and the presence of hypertension or dyslipidemia, and interactions between subgroups were tested. Statistical significance was defined as a two-sided P value < 0.05. All statistical analyses were performed using SAS version 9.4 (SAS Institute, Inc.) and R version 3.2.4 (R Core Team) software.

## Results

### Baseline characteristics of study population

The baseline characteristics of a total of 139,633 patients with T2DM according to FLI cutoff value are summarized in Table 1. The NAFLD group defined as FLI ≥ 60 consisted of 47,146 (34%) patients, the intermediate group defined as 30 ≤ FLI < 60 consisted of 49,408 (35%) patients, and the no NAFLD group defined as FLI < 30 consisted of 43,079 (31%) patients. The NAFLD patients were younger, more likely to be male and mild drinkers, and more likely to have higher baseline BMI, waist circumference, systolic blood pressure (BP), diastolic BP, fasting glucose, AST, ALT, gamma-glutamyl transferase, and triglycerides. Hypertension and dyslipidemia were more frequent in the NAFLD group. Insulin users were most common in the group without NAFLD, while those who did not take oral hypoglycemic agents were least prevalent in this group.

**Table 1:** Baseline characteristics of the study population according to FLI score (*n* = 139,633)

	FLI < 30 (*n* = 43,079)	30 ≤ FLI < 60 (*n* = 49,408)	FLI ≥ 60 (*n* = 47,146)	*p*-value
Age (years)	57.7 ± 11.5	57.0 ± 10.7	52.8 ± 11.0	< 0.001
Men	16,881 (39.2)	25,982 (52.6)	31,982 (67.8)	< 0.001
Income level, lowest 20%	7743 (18.0)	8300 (16.8)	7777 (16.5)	< 0.001
Smoking				< 0.001
Never	30,502 (70.8)	29,937 (60.6)	22,349 (47.4)	
Former	5218 (12.1)	8496 (17.2)	9629 (20.4)	
Current	7359 (17.1)	10,975 (22.2)	15,168 (32.2)	
Mild drinker	12,485 (29.0)	18,716 (37.9)	24,271 (51.5)	< 0.001
Regular exercise	7969 (18.5)	8675 (17.6)	7118 (15.1)	< 0.001
Insulin user	3487 (8.09)	2647 (5.36)	2392 (5.07)	< 0.001
Oral hypoglycemic agent				< 0.001
None	2140 (5.0)	1318 (2.7)	1062 (2.3)	
Single	28,684 (66.6)	33,250 (67.3)	30,917 (65.6)	
Dual combination	11,515 (26.7)	14,103 (28.5)	14,403 (30.6)	
Triple combination	740 (1.7)	737 (1.5)	764 (1.6)	
BMI (kg/m^2^)	22.8 ± 2.3	25.3 ± 2.3	28.1 ± 3.4	< 0.001
Waist circumference (cm)				
In men	79.8 ± 5.8	86.0 ± 5.2	92.6 ± 8.2	< 0.001
In women	77.3 ± 6.1	84.8 ± 5.8	92.8 ± 9.7	< 0.001
SBP (mmHg)	126.2 ± 16.3	129.7 ± 16.1	132.4 ± 16.2	< 0.001
DBP (mmHg)	77.6 ± 10.1	80.1 ± 10.2	82.7 ± 10.7	< 0.001
Fasting plasma glucose (mg/dl)	161.7 ± 66.1	165.2 ± 61.5	169.0 ± 59.6	< 0.001
AST (IU/L)	21.7 (21.6–21.8)	25.7 (25.6–25.8)	33.6 (33.4–33.7)	< 0.001
ALT (IU/L)	20.5 (20.5–20.6)	28.5 (28.4–28.6)	41.6 (41.4–41.9)	< 0.001
GGT (IU/L)	23.7 (23.6–23.8)	39.34 (39.2–39.6)	71.7 (71.2–72.1)	< 0.001
Total cholesterol (mg/dl)	207.5 ± 43.1	216.8 ± 44.8	225.0 ± 52.4	< 0.001
Triglycerides (mg/dl)	106.0 (105.6–106.5)	162.4 (161.7–163.0)	241.4 (240.2–242.5)	< 0.001
HDL-C (mg/dl)	55.7 ± 24.0	51.8 ± 23.5	50.0 ± 54.3	< 0.001
LDL-C (mg/dl)	129.5 ± 56.4	131.0 ± 53.2	124.1 ± 92.1	< 0.001
eGFR (ml/min/1.73 m^2^)	89.0 ± 36.8	87.0 ± 32.1	87.8 ± 39.5	< 0.001
Hypertension	18,227 (42.3)	25,616 (51.9)	27,578 (58.5)	< 0.001
Dyslipidemia	16,664 (38.7)	23,864 (48.3)	26,085 (55.3)	< 0.001

Continuous variables are expressed as mean ± standard deviation or median (interquartile range)

Categorical data are presented as frequencies and percentages

AST, alanine aminotransferase; ALT, aspartate aminotransferase; BMI, body mass index; DBP, diastolic blood pressure; eGFR, estimated glomerular filtration rate; FLI, fatty liver index; GGT, gamma-glutamyl transferase; HDL-C, high-density lipoprotein cholesterol; LDL-C, low-density lipoprotein cholesterol; SBP, systolic blood pressure

### Hepatic steatosis based on FLI and the CV outcomes and mortality

During the median follow-up of 7.7 years, there were 3079 (2.2%) cases of MI, 4238 (3.0%) cases of ischemic stroke, 4303 (3.1%) cases of HF, and 8465 (6.1%) all-cause deaths. Table 2 shows the results of multivariate Cox regression analysis: hepatic steatosis (FLI ≥ 60) was associated with increased risk for development of MI (adjusted HR [aHR] 1.28, 95% CI [1.14–1.44]), ischemic stroke (1.41 [1.25–1.56]), heart failure (1.17 [1.07–1.26]), and mortality (1.41 [1.32–1.51]) after adjusting for age, sex, alcohol consumption, smoking, regular exercise, income status, body weight, hypertension, dyslipidemia, fasting glucose, and number of oral hypoglycemic agents used. There was a gradual association between higher FLI values and greater incidence of CV outcomes including myocardial infarction, ischemic stroke, heart failure, and mortality (all *p* for trends < 0.001).

**Table 2:** Risk of myocardial infarction, stroke, heart failure, and mortality according to fatty liver index

	Event	Duration (person-years)	Incidence Rate^a^	Hazard Ratio (95% CI)	
				Model 1	Model 2
Myocardial infarction					
FLI < 30	898	325,568	2.76	1 (Ref)	1 (Ref)
30 ≤ FLI < 60	1110	379,063	2.93	1.05 (0.96–1.14)	1.08 (0.99–1.19)
FLI ≥ 60	1071	361,981	2.96	1.21 (1.10–1.33)	1.28 (1.14–1.44)
*P* for trend				< 0.001	< 0.001
Stroke					
FLI < 30	1305	324,031	4.03	1 (Ref)	1 (Ref)
30 ≤ FLI < 60	1626	377,182	4.31	1.10 (1.03–1.19)	1.22 (1.13–1.33)
FLI ≥ 60	1307	360,875	3.62	1.17 (1.08–1.26)	1.41 (1.28–1.56)
*P* for trend				< 0.001	< 0.001
Heart failure					
FLI < 30	2030	324,207	6.26	1 (Ref)	1 (Ref)
30 ≤ FLI < 60	2097	378,517	5.54	0.92 (0.86–0.97)	0.93 (0.87–0.99)
FLI ≥ 60	1976	361,310	5.47	1.16 (1.09–1.24)	1.17 (1.07–1.26)
*P* for trend				< 0.001	< 0.001
Mortality					
FLI < 30	3369	328,244	10.26	1 (Ref)	1 (Ref)
30 ≤ FLI < 60	2822	382,618	7.38	0.74 (0.70–0.78)	1.02 (0.96–1.07)
FLI ≥ 60	2274	365,317	6.22	0.81 (0.77–0.86)	1.41 (1.32–1.51)
*P* for trend				< 0.001	< 0.001

Model 1: Adjusted for age and sex

Model 2: Adjusted for age, sex, smoking status, alcohol consumption, regular exercise, income, body weight, hypertension, dyslipidemia, fasting glucose, and number of oral hypoglycemic agents used

CI, confidence interval; FLI, fatty liver index

^a^Incidence per 1000 person-years

### Hepatic fibrosis based on BARD score and the CV outcomes and mortality

Among 47,146 patients with hepatic steatosis (defined as FLI ≥ 60), 23,321 (49.0%) had advanced fibrosis (defined as BARD ≥ 2). There were 1071 (2.3%) cases of MI, 1307 (2.8%) cases of ischemic stroke, 1976 (4.2%) case of HF, and 2274 (4.8%) all-cause deaths in patients with NAFLD during the follow-up period. Compared to the group without NAFLD (FLI < 60), the group with hepatic steatosis and without fibrosis (FLI ≥ 60 and BARD < 2) and the group with both hepatic steatosis and fibrosis (FLI ≥ 60 and BARD ≥ 2) showed gradual increased risk for MI (aHR [95% CI], 1.26 [1.14–1.40]), ischemic stroke (1.26 [1.15–1.37]), heart failure ([1.22–1.41]), and all-cause mortality (1.48 [1.39–1.58]) in the fully-adjusted model (all *p* for trends < 0.001, Table 3). The aHRs and 95% CIs for CVD and mortality according to BARD score in both FLI < 60 and FLI ≥ 60 compared to FLI < 60 and BARD < 2 are presented in electronic supplementary material [ESM] Table 1. The aHRs and 95% CIs for CVD and mortality the group with advanced fibrosis (BARD ≥ 2) compared to those with BARD < 2 in FLI ≥ 60 are indicated in electronic supplementary material [ESM] Table 2.

**Table 3:** Risk of myocardial infarction, stroke, heart failure, and mortality according to BARD score in patients with fatty liver index (FLI) ≥ 60 compared to FLI < 60

	Event	Duration (person-years)	Incidence Rate^a^	Hazard Ratio (95% CI)	
				Model 1	Model 2
Myocardial infarction					
FLI < 60	2008	704,631	2.85	1 (Ref)	1 (Ref)
FLI ≥ 60, BARD < 2	457	184,258	2.48	1.13 (1.02–1.26)	1.13 (1.00–1.27)
FLI ≥ 60, BARD ≥ 2	614	177,722	3.45	1.21 (1.11–1.33)	1.26 (1.14–1.40)
*P* for trend				< 0.001	< 0.001
Stroke					
FLI < 60	2931	701,213	4.18	1 (Ref)	1 (Ref)
FLI ≥ 60, BARD < 2	490	184,039	2.66	1.02 (0.92–1.12)	1.13 (1.01–1.26)
FLI ≥ 60, BARD ≥ 2	817	176,836	4.62	1.16 (1.07–1.25)	1.26 (1.15–1.37)
*P* for trend				< 0.001	< 0.001
Heart failure					
FLI < 60	4127	702,724	5.87	1 (Ref)	1 (Ref)
FLI ≥ 60, BARD < 2	724	184,297	3.93	1.11 (1.02–1.20)	1.10 (1.01–1.21)
FLI ≥ 60, BARD ≥ 2	1252	177,013	7.07	1.29 1.21–1.38)	1.31 (1.22–1.41)
*P* for trend				< 0.001	< 0.001
Mortality					
FLI < 60	6191	710,862	8.71	1 (Ref)	1 (Ref)
FLI ≥ 60, BARD < 2	741	185,750	3.99	0.81 (0.75–0.87)	1.22 (1.23–1.33)
FLI ≥ 60, BARD ≥ 2	1533	179,567	8.54	1.03 (0.97–1.09)	1.48 (1.39–1.58)
*P* for trend				0.736	< 0.001

Model 1: Adjusted for age and sex

Model 2: Adjusted for age, sex, smoking status, alcohol consumption, regular exercise, income, body weight, hypertension, dyslipidemia, fasting glucose, and number of oral hypoglycemic agents used

CI, confidence interval

^a^Incidence per 1000 person-years

### Subgroup analysis

We conducted subgroup analyses stratified by age, sex, BMI, hypertension, and dyslipidemia. The aHR and 95% CI values for all-cause death in NAFLD (FLI ≥ 60) compared to those without NAFLD (FLI < 30) according to these subgroups are presented in Fig. 1. In subgroup analyses, the positive association between NAFLD and all-cause mortality was consistent, but there was an interaction between the age and dyslipidemia subgroups. In addition, subgroup analyses for MI, stroke, and HF are shown in electronic supplementary material [ESM] Table 3. NAFLD showed a positive association with MI and stroke in all subgroups analyzed. NAFLD was associated with HF in subgroups stratified by age, hypertension, and dyslipidemia. In the group with BMI < 25 kg/m^2^, although the association of NAFLD with heart failure was slightly attenuated, there was no interaction between the two groups. However, in subgroup analysis stratified by sex, the association between NAFLD and heart failure was significant only in the female subgroup.

## Discussion

In this population-based longitudinal study with a median follow-up period of eight years, hepatic steatosis assessed by FLI was associated with increased risk of MI, ischemic stroke, HF, and mortality in newly diagnosed T2DM. Moreover, we demonstrated that advanced hepatic fibrosis assessed by BARD score was significantly associated with increased risk of CV outcomes and mortality in patients with new-onset T2DM. These associations remained significant after adjusting for other covariates such as body weight, hypertension, dyslipidemia, and number of oral hypoglycemic agents used. To our knowledge, this is the first cohort study to evaluate the longitudinal associations between hepatic steatosis and/or fibrosis and risk of CVD and mortality in new-onset T2DM.

NAFLD is considered a CV risk factor independent of established atherosclerotic risk factors and features of metabolic syndrome, and CVD in NAFLD is a leading cause of death [3, 7, 8, 21]. The mechanism linking NAFLD and CVD is explained by insulin resistance, oxidative stress, inflammation, endothelial dysfunction, and altered lipid metabolism [2, 7, 22]. The majority of studies evaluating NAFLD and CVD has included subjects from the general population, encompassing individuals with or without diabetes, not focusing on patients with diabetes. Therefore, few studies have demonstrated the risk of CVD that NAFLD poses in patients with diabetes. One observational study reported the association between NAFLD diagnosed by ultrasonography (US) and CVD including MI, ischemic stroke, and CV death in 2103 T2DM patients [23]. Another reported that US-confirmed hepatic steatosis and biomarker-based hepatic fibrosis increased the progression of carotid atherosclerosis in 1120 T2DM patients [24]. However, these studies are based on a single center and small population with diabetes duration of longer than five years or 10 years. In contrast, our study targeted newly diagnosed T2DM to minimize the impact of diabetes, which is the strong and potential CV risk factor in evaluating the association between NAFLD and CVD in T2DM; therefore, we demonstrated that NAFLD could contribute additional cardiovascular risk in T2DM. In addition, we used the FLI as a well-established prediction model for hepatic steatosis; therefore, we identified the associations between hepatic steatosis and CVD and mortality in a large nationwide population. T2DM is more susceptible to advanced hepatic fibrosis, and hepatic fibrosis increased CV risk in patients with hepatic steatosis [25]. Our study also showed that advanced hepatic fibrosis, defined by a BARD score of at least two points, was associated with increased risk for CVD and mortality among new-onset T2DM patients with NAFLD. This means that presence of not only steatosis, but also fibrosis was progressively associated with CVD and mortality in new-onset T2DM. Because our entire study population had diabetes, the presence of additional components, either BMI ≥ 28 kg/m^2^ or AST/ALT ratio ≥ 0.8, determined the presence of hepatic fibrosis. However, the association between hepatic fibrosis assessed by BARD score and HF and mortality in new-onset T2DM was not different compared to our previous study of the general population [10]. In this line, we found that hepatic steatosis and/or fibrosis are associated not only with HF and mortality, but also with MI and ischemic stroke in new-onset T2DM.

Obesity and insulin resistance play a crucial pathogenic role in both NAFLD and T2DM; therefore, an improvement in one can affect outcomes of the other. Although there are no specific FDA-approved drug options available for treatment for NAFLD or steatohepatitis, and a lifestyle change toward healthy diet and physical activity is crucial and fundamental for management of NAFLD as well as T2DM, the importance of assessment for NAFLD at diagnosis of T2DM has been underestimated. Relative to lifestyle modification, pharmacologic treatment for diabetes is more powerful, and some anti-diabetes medication showed additional beneficial effects on resolution of hepatic fat accumulation and reduction of risk factors associated with CVD when added to lifestyle modification [26]. Treatment with pioglitazone, one of the thiazolidinediones, for 18 months in patients with prediabetes or T2DM led to marked improvement in hepatic steatosis, inflammation, and ballooning and reduced hepatic fibrosis progression compared to the placebo group [27]. Of note, the placebo group showed a relatively high rate of hepatic fibrosis within 3 years, consistent with a previous study that reported that NAFLD could progress to NASH within 5 years, especially if metabolic risk factors deteriorate [28]. In addition, treatment with sodium-glucose cotransporter 2 (SGLT-2) inhibitors such as dapagliflozin or empagliflozin was reported to reduce liver fat content in T2DM [29, 30]. CVD is a major diabetic complication and the leading cause of mortality in patients with diabetes. Therefore, it has been recommended that proactive pharmacologic therapy is necessary for diabetes management in patients with diabetes combined with advanced liver disease or in high-risk groups for hepatic disease progression [31]. In line with our study, in terms of risk stratification for CVD outcomes, determining the presence of hepatic steatosis and/or fibrosis assessed by FLI or BARD score might have an additive role for management of new-onset T2DM.

The strength of this study is its longitudinal and large population-based characteristics over a median follow-up of 8 years. In addition, it is the first to evaluate the associations between hepatic steatosis and/or fibrosis using well-validated prediction models and CVD and mortality in new-onset T2DM patients who are known to be susceptible to adverse hepatic and CVD outcomes. While a few other studies have evaluated the associations between NAFLD and CVD in T2DM patients included as a subset of their study populations, our large-scale longitudinal study differed in that it: (1) included only newly-diagnosed T2DM patients to evaluate the causal relationships of NAFLD, and (2) considered including oral hypoglycemic agents as a covariate in multivariate analysis. Therefore, we demonstrated that NAFLD itself could contribute additional cardiovascular risk in such patients. However, this study has several limitations. First, we used a biochemical scoring model in the diagnosis of hepatic steatosis and/or fibrosis rather than liver biopsy, which is considered as the gold-standard for diagnosis, or imaging such as ultrasonography, due to the lack of the data. Therefore, we could not determine the correlation of FLI or BARD with the extent of hepatic steatosis or fibrosis on ultrasonography. The Korean NHIS database did not include platelet count or albumin level, so we could not evaluate other fibrosis scores such as FIB-4 or the NAFLD fibrosis score. However, because FLI and BARD score are well validated worldwide [18, 19, 32, 33], and the association between FLI or BARD score and CV outcomes in the general population has been reported in several studies [9, 10], these prediction models could be also used in prediction of the risk for CVD in our study population. Second, CVD diagnosis based on ICD-10 codes might be under- or over-estimated. Third, any change in the oral hypoglycemic agents used during the follow-up period could not be considered. Fourth, hemoglobin A1c was not available in the NHIS database, and we could not assess diabetes control in the study population. Fifth, our study included only a Korean population, limiting generalization to other ethnic groups.

## Conclusion

In conclusion, advanced liver fibrosis assessed by BARD score as well as hepatic steatosis assessed by FLI were associated with increased risk of MI, ischemic stroke, HF, and mortality in new-onset T2DM. This study offers compelling evidence that not only hepatic steatosis but also advanced liver fibrosis should be assessed and closely monitored from the time of diagnosis of diabetes. Because proactive interventions such as healthy diet, physical activity, and pharmacotherapy may halt or reverse steatosis or advanced fibrosis and then CVD or mortality can also be prevented. This study gives strength to this hypothesis, and further investigation is needed in the future study.

## Supplementary Information

**Additional file 1: Table S1.** Risk of myocardial infarction, stroke, heart failure, and mortality according to FLI and BARD score in both FLI < 60 and FLI ≥ 60. **Table S2.** Risk of myocardial infarction, stroke, heart failure, and mortality according to BARD score in patients with fatty liver index (FLI) ≥ 60. **Table S3.** Subgroup analysis for myocardial infarction, heart failure, and stroke in patients with fatty liver index ≥ 60 compared to fatty liver index < 30. **Figure S1.** Study flow.

## Data Availability

The data that support the findings of this study are available form Korean National Health Insurance Service (KNHIS), but restrictions apply to their availability. However, data are available from the authors upon reasonable request and with permission from the KNHIS.

## Abbreviations

BMI: Body mass index
CI: Confidence interval
CVD: Cardiovascular disease
FLI: Fatty liver index
HF: Heart failure
HR: Hazard ratio
NAFLD: Nonalcoholic fatty liver disease
MI: Myocardial infarction
