# Supplementary material for: The associations of hepatic steatosis and fibrosis using fatty liver index and BARD score with cardiovascular outcomes and mortality in patients with new-onset type 2 diabetes: a nationwide cohort study
Source: Cardiovasc Diabetol. 2022 Apr 16;21:53. doi: 10.1186/s12933-022-01483-y (PMC9013458; doi:10.1186/s12933-022-01483-y)
Supplement: Supplementary file 1 — Additional file 1: Table S1. Risk of myocardial infarction, stroke, heart failure, and mortality according to FLI and BARD score in both FLI < 60 and FLI ≥ 60. Table S2. Risk of myocardial infarction, stroke, heart failure, and mortality according to BARD score in patients with fatty liver index (FLI) ≥ 60. Table S3. Subgroup analysis for myocardial infarction, heart failure, and stroke in patients with fatty liver index ≥ 60 compared to fatty liver index < 30. Figure S1. Study flow. [file 12933_2022_1483_MOESM1_ESM.docx]

**Additional file 1**

**The Associations of Hepatic Steatosis and Fibrosis Using Fatty Liver Index and BARD Score with Cardiovascular Outcomes and Mortality in Patients with New-Onset Type 2 Diabetes: A Nationwide Cohort Study**

Jiyun Park^1†^, Gyuri Kim^1†^, Bong-Sung Kim^2^, Kyung-Do Han^2*^, So Yoon Kwon^1^, So Hee Park^1^, You-Bin Lee^1^, Sang-Man Jin^1^, Jae Hyeon Kim^1,3*^

^1^Division of Endocrinology and Metabolism, Department of Medicine, Samsung Medical Center, Sungkyunkwan University School of Medicine, Seoul, Republic of Korea

^2^Department of Statistics and Actuarial Science, Soongsil University, Seoul, Republic of Korea

^3^Department of Clinical Research Design and Evaluation, Samsung Advanced Institute for

Health Sciences and Technology, Seoul, Republic of Korea

^†^These two authors contributed equally.

^*^**Correspondence:**

Jae Hyeon Kim, MD, PhD

Division of Endocrinology and Metabolism, Department of Medicine, Samsung Medical Center, Sungkyunkwan University School of Medicine

81, Irwon-ro, Gangnam-gu, Seoul, 06351, Republic of Korea

Email: jaehyeon@skku.edu, Phone: +82-2-3410-1580

Kyungdo Han, PhD

Department of Statistics and Actuarial Science, Soongsil University

369 Sangdo-ro, Dongjak-gu, Seoul, 06978, Republic of Korea

Email: hkd917@naver.com, Phone: +82-2-828-7025

**Table S1.** Risk of myocardial infarction, stroke, heart failure, and mortality according to FLI and BARD score in both FLI < 60 and FLI ≥ 60

|  | **Event** | **Incidence Rate^*^** | **Hazard ratio (95% CI)** | |
| --- | --- | --- | --- | --- |
|  |  |  | **Model 1** | **Model 2** |
| **Myocardial infarction** |  |  |  |  |
| FLI < 60/BARD < 2 | 503 | 2.56 | 1 (Ref) | 1 (Ref) |
| FLI < 60/BARD ≥ 2 | 1505 | 2.96 | 0.96 (0.86-1.06) | 0.98 (0.88-1.09) |
| FLI ≥ 60/BARD < 2 | 457 | 2.48 | 1.10 (0.96-1.24) | 1.11 (0.97-1.28) |
| FLI ≥ 60/BARD ≥ 2 | 614 | 3.45 | 1.17 (1.04-1.32) | 1.24 (1.10-1.41) |
| **Stroke** |  |  |  |  |
| FLI < 60/BARD < 2 | 623 | 3.18 | 1 (Ref) | 1 (Ref) |
| FLI < 60/BARD ≥ 2 | 2308 | 4.57 | 1.03 (0.94-1.13) | 1.03 (0.94-1.12) |
| FLI ≥ 60/BARD < 2 | 490 | 2.66 | 1.04 (0.93-1.17) | 1.15 (1.01-1.30) |
| FLI ≥ 60/BARD ≥ 2 | 817 | 4.62 | 1.19 (1.07-1.32) | 1.28 (1.15-1.43) |
| **Heart failure** |  |  |  |  |
| FLI < 60/BARD < 2 | 804 | 4.09 | 1 (Ref) | 1 (Ref) |
| FLI < 60/BARD ≥ 2 | 3323 | 6.56 | 1.16 (1.07-1.25) | 1.17 (1.08-1.27) |
| FLI ≥ 60/BARD < 2 | 724 | 3.93 | 1.23 (1.12-1.37) | 1.23 (1.11-1.37) |
| FLI ≥ 60/BARD ≥ 2 | 1252 | 7.07 | 1.45 (1.33-1.58) | 1.48 (1.35-1.62) |
| **Mortality** |  |  |  |  |
| FLI < 60/BARD < 2 | 961 | 4.85 | 1 (Ref) | 1 (Ref) |
| FLI < 60/BARD ≥ 2 | 5230 | 10.20 | 1.40 (1.30-1.50) | 1.28 (1.19-1.37) |
| FLI ≥ 60/BARD < 2 | 23825 | 3.99 | 1.04 (0.95-1.15) | 1.47 (1.33-1.62) |
| FLI ≥ 60/BARD ≥ 2 | 23321 | 8.54 | 1.34 (1.24-1.46) | 1.80 (1.65-1.96) |

Model 1: adjusted for age and sex.

Model 2: adjusted for age, sex, smoking status, alcohol consumption, regular exercise, income, body weight, hypertension, dyslipidemia, fasting glucose, and number of oral hypoglycemic agents used.

^*^incidence per 1,000 person-years.

CI, confidence interval

**Table S2.** Risk of myocardial infarction, stroke, heart failure, and mortality according to BARD score in patients with fatty liver index (FLI) ≥ 60

|  | **Event** | **Duration**  **(person**  **-years)** | **Incidence Rate^a^** | **Hazard Ratio (95% CI)** | |
| --- | --- | --- | --- | --- | --- |
|  |  |  |  | **Model 1** | **Model 2** |
| **Myocardial infarction** |  |  |  |  |  |
| BARD < 2 | 457 | 184,258 | 2.48 | 1 (Ref) | 1 (Ref) |
| BARD ≥ 2 | 614 | 177,722 | 3.45 | 1.12 (0.99-1.28) | 1.17 (1.03-1.33) |
| **Stroke** |  |  |  |  |  |
| BARD < 2 | 490 | 184,039 | 2.66 | 1 (Ref) | 1 (Ref) |
| BARD ≥ 2 | 817 | 176,836 | 4.62 | 1.14 (1.01-1.28) | 1.11 (0.99-1.25) |
| **Heart failure** |  |  |  |  |  |
| BARD < 2 | 724 | 184,297 | 3.93 | 1 (Ref) | 1 (Ref) |
| BARD ≥ 2 | 1252 | 177,013 | 7.07 | 1.21 (1.10-1.33) | 1.25 (1.14-1.38) |
| **Mortality** |  |  |  |  |  |
| BARD < 2 | 741 | 185,750 | 3.99 | 1 (Ref) | 1 (Ref) |
| BARD ≥ 2 | 1533 | 179,567 | 8.54 | 1.37 (1.25-1.50) | 1.34 (1.22-1.46) |

Model 1: Adjusted for age and sex.

Model 2: Adjusted for age, sex, smoking status, alcohol consumption, regular exercise, income, body weight, hypertension, dyslipidemia, fasting glucose, and number of oral hypoglycemic agents used.

^a^Incidence per 1,000 person-years.

CI, confidence interval

**Table S3.** Subgroup analysis for myocardial infarction, heart failure, and stroke in patients with fatty liver index ≥ 60 compared to fatty liver index < 30

|  | **Hazard Ratio (95% Confidence Interval)** | | |
| --- | --- | --- | --- |
|  | **Myocardial infarction** | **Stroke** | **Heart failure** |
| **Age** |  |  |  |
| < 65 (*n =* 70,952) | 1.45 (1.25-1.68) | 1.34 (1.20-1.57) | 1.23 (1.10-1.37) |
| ≥ 65 (*n =* 19,273) | 1.19 (1.01-1.40) | 1.44 (1.26-1.64) | 1.13 (1.02-1.26) |
| *P* for interaction | 0.040 | 0.531 | 0.194 |
| **Sex** |  |  |  |
| Male (*n =* 48,863) | 1.33 (1.15-1.54) | 1.34 (1.18-1.52) | 1.10 (0.99-1.22) |
| Female (*n =* 41,362) | 1.32 (1.12-1.56) | 1.48 (1.29-1.70) | 1.26 (1.23-1.40) |
| *P* for interaction | 0.945 | 0.219 | 0.041 |
| **Body mass index** |  |  |  |
| < 25 (*n =* 43,237) | 1.27 (1.07-1.50) | 1.39 (1.20-1.60) | 1.11 (0.98-1.26) |
| ≥ 25 (*n =* 46,988) | 1.36 (1.11-1.67) | 1.42 (1.20-1.69) | 1.22 (1.07-1.39) |
| *P* for interaction | 0.557 | 0.813 | 0.272 |
| **Hypertension** |  |  |  |
| No (*n* = 44,420) | 1.44 (1.22-1.70) | 1.36 (1.16-1.58) | 1.18 (1.04-1.33) |
| Yes (*n =* 45,805) | 1.25 (1.09-1.45) | 1.42 (1.26-1.60) | 1.17 (1.06-1.30) |
| *P* for interaction | 0.132 | 0.571 | 0.981 |
| **Dyslipidemia** |  |  |  |
| No (*n =* 47,476) | 1.29 (1.11-1.50) | 1.39 (1.22-1.59) | 1.11 (1.00-1.23) |
| Yes (*n =* 42,749) | 1.37 (1.17-1.59) | 1.41 (1.23-1.61) | 1.24 (1.12-1.39) |
| *P* for interaction | 0.535 | 0.888 | 0.074 |

**Figure S1.** Study flow
